# Protective Effects of Testosterone on Presynaptic Terminals against Oligomeric **β**-Amyloid Peptide in Primary Culture of Hippocampal Neurons

**DOI:** 10.1155/2014/103906

**Published:** 2014-06-18

**Authors:** Chi-Fai Lau, Yuen-Shan Ho, Clara Hiu-Ling Hung, Suthicha Wuwongse, Chun-Hei Poon, Kin Chiu, Xifei Yang, Leung-Wing Chu, Raymond Chuen-Chung Chang

**Affiliations:** ^1^Laboratory of Neurodegenerative Diseases, Department of Anatomy, LKS Faculty of Medicine, Room L1-49, 21 Sassoon Road, Pokfulam, Hong Kong; ^2^State Key Laboratory of Quality Research in Chinese Medicine, Macau University of Science and Technology, Avenida Wai Long, Taipa, Macau; ^3^Institute of Chinese Medicinal Science, University of Macau, Macau; ^4^Department of Psychiatry, LKS Faculty of Medicine, Hong Kong; ^5^Department of Ophthalmology, LKS Faculty of Medicine, Hong Kong; ^6^Shenzhen Centre for Disease Control and Prevention, Shenzhen, China; ^7^Division of Geriatric, Department of Medicine, LKS Faculty of Medicine, The University of Hong Kong, Pokfulam, SAR, Hong Kong; ^8^Research Centre of Heart, Brain, Hormone and Healthy Aging, LKS Faculty of Medicine, Hong Kong; ^9^State Key Laboratory of Brain and Cognitive Sciences, Hong Kong

## Abstract

Increasing lines of evidence support that testosterone may have neuroprotective effects. While observational studies reported an association between higher bioavailable testosterone or brain testosterone levels and reduced risk of Alzheimer's disease (AD), there is limited understanding of the underlying neuroprotective mechanisms. Previous studies demonstrated that testosterone could alleviate neurotoxicity induced by *β*-amyloid (A*β*), but these findings mainly focused on neuronal apoptosis. Since synaptic dysfunction and degeneration are early events during the pathogenesis of AD, we aim to investigate the effects of testosterone on oligomeric A*β*-induced synaptic changes. Our data suggested that exposure of primary cultured hippocampal neurons to oligomeric A*β* could reduce the length of neurites and decrease the expression of presynaptic proteins including synaptophysin, synaptotagmin, and synapsin-1. A*β* also disrupted synaptic vesicle recycling and protein folding machinery. Testosterone preserved the integrity of neurites and the expression of presynaptic proteins. It also attenuated A*β*-induced impairment of synaptic exocytosis. By using letrozole as an aromatase antagonist, we further demonstrated that the effects of testosterone on exocytosis were unlikely to be mediated through the estrogen receptor pathway. Furthermore, we showed that testosterone could attenuate A*β*-induced reduction of HSP70, which suggests a novel mechanism that links testosterone and its protective function on A*β*-induced synaptic damage. Taken together, our data provide further evidence on the beneficial effects of testosterone, which may be useful for future drug development for AD.

## 1. Introduction

Cognitive dysfunction or impairment is the major symptom in Alzheimer's disease (AD). Loss of synaptic proteins leading to synaptic dysfunctions and perturbation of cytoskeleton leading to disturbance of axonal transport may explain the underlying mechanisms of these cognitive changes [1, 2]. Neurotransmission is a complicated process which involves multiple steps: synthesis and storage of neurotransmitter in synaptic vesicles, transmission of synaptic vesicles towards presynaptic density membrane, docking of synaptic vesicles to presynaptic membrane, release of neurotransmitter, reuptake of excessive neurotransmitters, and recycling of synaptic vesicles [3]. These processes must be highly regulated. Dysfunction of any of the above steps will impair normal neurotransmission, which is indeed a major pathological change during the progression of AD [4–6]. It has been shown that oligomeric *β*-amyloid (A*β*) peptide can impair both presynaptic and postsynaptic density proteins and receptors for neurotransmitters in experimental models of AD [7–10]. Preventing the deterioration of synaptic degeneration is therefore an important therapeutic approach to delay the progression of AD.

The reduction of bioavailable testosterone is associated with increased levels of A*β* peptides, hyperphosphorylation of tau protein, and neuronal cell death. Therefore, the loss of bioavailable testosterone and its metabolites dihydrotestosterone or estrogen has been suggested as a risk factor of developing dementia and even AD [11–14]. The significance of testosterone in the development of AD has been extensively investigated by Pike's laboratory. They reported that androgen regulates A*β* levels via androgen receptor (AR) and estrogen receptor (ER) in cell cultures and rodent AD models [15]. Testosterone had been shown to increase neuronal viability in cultured hippocampal neurons through the AR-dependent mitogen-activated protein kinase (MAPK)/extracellular signal related kinase (ERK) signaling pathway [16]. Testosterone could also elevate the levels of neprilysin to facilitate A*β* clearance [17]. Furthermore, it was found that the nonaromatizable androgen dihydrotestosterone (DHT), which is converted from testosterone, exerted neuroprotective effects by activating the AR-dependent cyclic AMP response element binding protein (CREB) signaling pathway in PC12 cells and cultured hippocampal neurons [18]. Evidence in AD mice models showed that DHT elevated the levels of neprilysin and reduced A*β* levels via AR-independent pathway [19, 20]. A more recent finding further demonstrated that testosterone promoted the degradation of A*β* through an estrogenic pathway-independent manner [21]. All these data suggested that physiological level of testosterone is likely to reduce the chance of developing AD.

Preventing the loss or degeneration of synapse at early phase of AD is important [1, 22]. Since it has been suggested that testosterone is neuroprotective and prevents neurodegeneration, it is reasonable to speculate that testosterone may elicit a protective role in synapse. It has been well reported that synaptic vesicle proteins play a curial role in neurotransmission, and synaptic vesicle proteins recycling was decreased by oligomeric A*β* [9, 23]. Heat shock proteins are chaperones that help in preventing protein aggregation and fighting against cellular stress and thus have a potential implication for neurodegenerative disorders [24, 25]. HSP70 and HSP90 were found to suppress A*β* aggregation* in vitro* [26]. A study using human neurons reported that testosterone increased HSP70 protein levels and consequently attenuated A*β* toxicity [27]. In the present study, we aim to investigate whether testosterone can prevent synaptic degeneration triggered by oligomeric A*β* peptide. We demonstrate that physiological level of testosterone reversed A*β*-induced neurite damage, loss of synaptic vesicle proteins, and exocytosis dysfunction. The synaptoprotective effects of testosterone are accomplished by an ER-independent pathway.

## 2. Materials and Methods

### 2.1. Animals

Animal experimental protocol was approved by the Committee on the Use of Live Animals in Teaching and Research of The University of Hong Kong. The Laboratory Animal Unit of The University of Hong Kong is accredited by the Association for Assessment and Accreditation for Laboratory Animal Care (AAALAC International).

### 2.2. Primary Cultures of Rat Hippocampal Neurons

Primary cultures of rat hippocampal neurons were prepared from embryonic day 18 Sprague-Dawley rat embryos by using the method described previously [28]. Briefly, hippocampi were dissected in 1X PBS supplemented with glucose (18 mM). They were then mechanically dissociated in minimum essential medium (MEM) and then seeded onto poly-L-lysine (25 *μ*g/mL) coated 15 mm glass coverslips at density of 7 × 10^4^ cells/coverslip in neurobasal medium containing 2% B-27, glutamax (2 mM), penicillin (50 U/mL), and streptomycin (50 *μ*g/mL) (Gibco-BRL). Deoxyfluorouridine (dFUR) (Sigma) was added into the neuronal cultures at day* in vitro* 2 (DIV 2) at a final concentration of 1 *μ*M for inhibiting proliferating glial cells. Hippocampal neurons were cultured for 14 days at 37°C in a humidified 5% CO_2_ incubator prior to treatment. All treatments were performed in DIV 14 hippocampal neurons. We chose this developmental stage because it had been shown that neurons developed all synaptic components such as synaptic proteins and spine after 2 weeks of culture [29, 30].

### 2.3. Oligomeric A*β* Peptides Preparation and Treatment

Oligomeric A*β* was prepared according to our previous report [31]. A*β*_1–42_ peptide was dissolved in 1,1,1,3,3,3-hexafluoro-2-propanol (Sigma) and then it was dried overnight by air at room temperature. The peptide pellet was resuspended with anhydrous DMSO (Sigma) at 2 mM as final concentration. A*β* was then bath-sonicated for 30 min at room temperature. Aliquot A*β* peptide was stored at −80°C deep freezer before use. The working concentration of A*β* was 5 *μ*M in all experiments. This concentration of A*β* could induce synaptic degeneration including the reduction of synaptic proteins but not apoptosis when applied to hippocampal neurons for 24 h [28, 31].

To investigate the neurotoxicity of A*β* on primary cultures of hippocampal neurons, A*β* was diluted with NB medium and was added to the cell culture at DIV14 for 24 h. Neuroprotective effects of testosterone were investigated in pretreatment experiments. 10 nM of testosterone has been considered to be at physiological dose [32]. Previous reports showed that 10 nM testosterone elicited neuroprotective effects in primary neuronal cultures [33, 34]. Based on these findings, hippocampal neurons were incubated with 10 nM of testosterone (Sigma) for 1 h and then cotreated with oligomeric A*β* for 24 h. The control group was treated with DMSO as vehicle.

### 2.4. Immunocytochemistry

Immunocytochemical staining was performed according to our previous publication [35]. Primary cultures of hippocampal neurons were fixed with 4% paraformaldehyde for 20 min and then permeabilized with 0.1% Triton X-100 for 7 min at room temperature. Nonspecific binding of antibody was blocked with 5% bovine serum albumin (BSA) for 1 h. Neurons were incubated with primary antibodies MAP-2, synapsin-1 (1 : 400; Cell Signaling Technology), synaptophysin (1 : 400; Chemicon), synaptotagmin (1 : 400; Calbiochem), and HSP70 (1 : 400; Enzo life sciences) for 1 h. The neurons were washed with TBS and then incubated with secondary antibodies (Alexa-488; 1 : 400; Molecular Probes, Invitrogen) for 1 h at room temperature. Neurons were washed with TBS and finally mounted with ProLong Antifade Kit (Molecular Probes, Invitrogen). Immunostaining was analyzed using a Carl Zeiss LSM700 inverted confocal microscopy provided by Faculty Core Facility, HKU.

### 2.5. Detection of the Loading and Unloading Capability of Synaptic Vesicles by Using FM4-64 

The loading and unloading capacity of synaptic vesicles was determined by using FM4-64 probe (Molecular Probes, Invitrogen), which is a modified styryl dye widely used for the visualization of vacuoles and endocytic movement. The FM4-64 protocol was adopted and modified from previous publication [36]. For the measurement of synaptic vesicles loading capacity, FM probe (5 *μ*M) in HBSS was added to the neuronal cultures after drug treatment. Potassium chloride (100 mM) was added simultaneously to stimulate the neurons to induce endocytosis for 5 min. After rinsing with HBSS, the neurons were fixed with 4% paraformaldehyde and mounted with ProLong Antifade Kit and images were captured by Carl Zeiss LSM-510 Meta/Axiocam inverted confocal microscope. Similar procedures were carried out for the measurement of synaptic vesicles unloading capability. After rinsing with HBSS, the neurons were further incubated with potassium chloride (100 mM) for 5 min to induce complete exocytosis. The cultured neurons were then washed with HBSS to remove potassium chloride, fixed with 4% paraformaldehyde, and mounted and representative images were captured by the confocal microscope.

### 2.6. Statistical Analysis

One-way analysis of variance (ANOVA) was used to analyze the data for multiple variable comparisons. Student-Newman-Keuls test was used as a* post hoc* test. GraphPad Prism was used as the statistical software. Results were expressed in fold of control and are shown as mean ± standard error (SE) from at least three independent experiments. In each experiment, at least 5 fields were captured and 5 cells were counted in each treatment group.

## 3. Results

### 3.1. Testosterone Prevented Oligomeric A*β*-Induced Neuritic Damage and Synaptic Vesicle Proteins Disruption in Hippocampal Neurons

**Figure 1:**
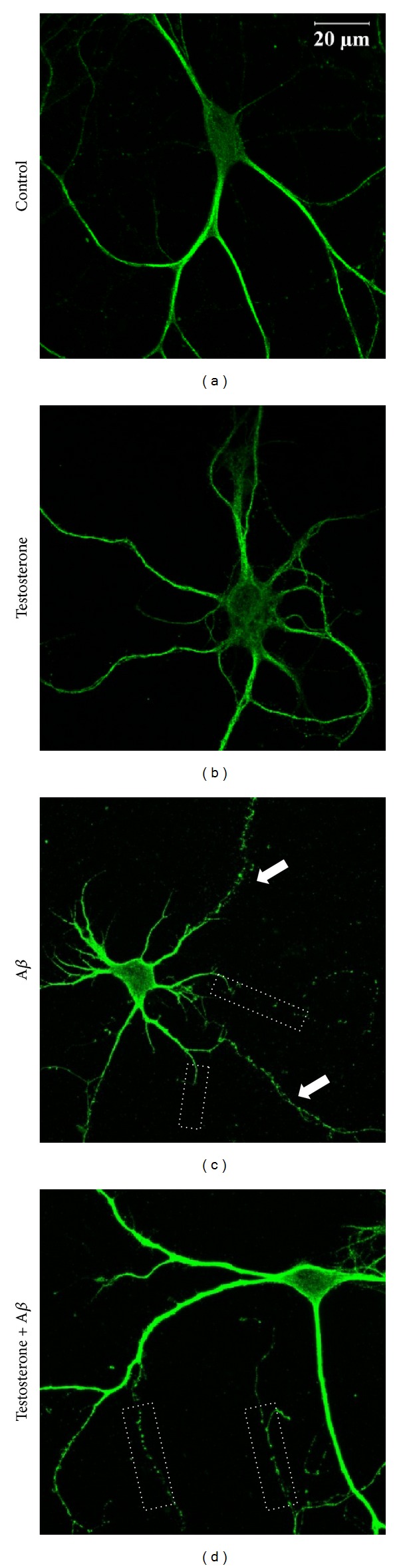
Testosterone reduced neurite shortening following oligomeric A*β* treatment in primary hippocampal neurons. Primary hippocampal neurons were treated with 10 nM testosterone for 1 h, followed by exposure to 5 *μ*M oligomeric A*β* for 24 h. Neurons were stained with MAP-2 antibody. (a) Control, (b) 10 nM testosterone for 25 h, (c) 5 *μ*M A*β* for 24 h, and (d) 10 nM testosterone for 1 h, followed by exposure to 5 *μ*M A*β* for 24 h. Arrows indicate fragmentation of neurites and white dot boxes indicate shortening of neurites.

**Figure 2:**
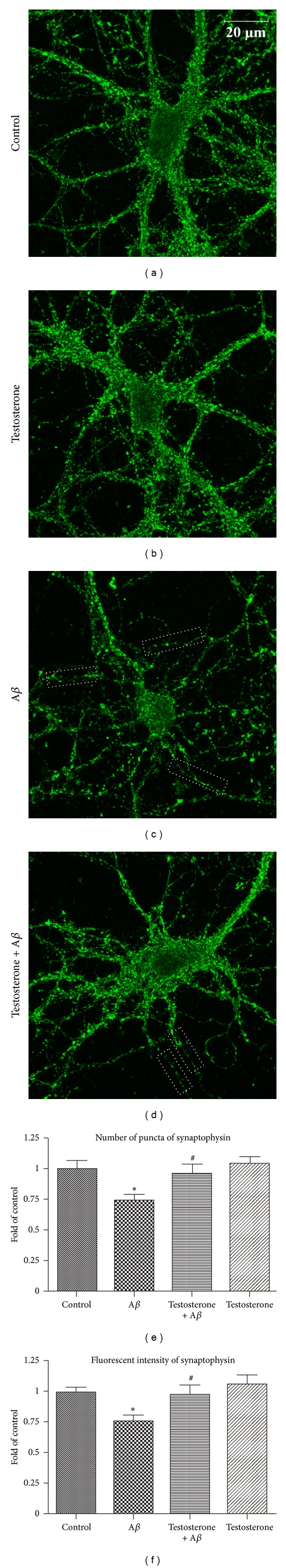
Testosterone attenuated oligomeric A*β*-induced reduction of synaptophysin in primary hippocampal neurons. Primary hippocampal neurons were treated with 10 nM testosterone for 1 h, followed by exposure to 5 *μ*M oligomeric A*β* for 24 h. Neurons were stained with synaptophysin antibody. (a) Control, (b) 10 nM testosterone for 25 h, (c) 5 *μ*M A*β* for 24 h, and (d) 10 nM testosterone for 1 h, followed by exposure to 5 *μ*M A*β* for 24 h. (e) The number of puncta and (f) the fluorescent intensity were analyzed by Image J software as described in the above section. *P < 0.05 versus control group, ^#^ *P* < 0.05 versus A*β* group. White dot boxes indicate reduced number of puncta along neurites in A*β*-treated group.

**Figure 3:**
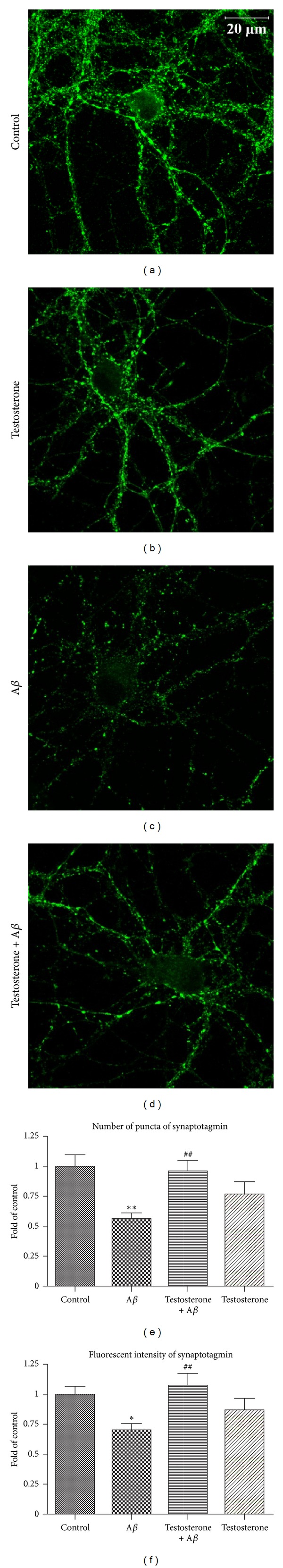
Testosterone attenuated oligomeric A*β*-induced reduction of synaptotagmin in primary hippocampal neurons. Primary hippocampal neurons were treated with 10 nM testosterone for 1 h, followed by exposure to 5 *μ*M oligomeric A*β* for 24 h. Neurons were stained with synaptotagmin antibody. (a) Control, (b) 10 nM testosterone for 25 h, (c) 5 *μ*M A*β* for 24 h, and (d) 10 nM testosterone for 1 h, followed by exposure to 5 *μ*M A*β* for 24 h. (e) The number of puncta and (f) the fluorescent intensity were analyzed by Image J software as described in the above section. *P < 0.05 versus control group, **P < 0.005 versus control group, and ^##^ *P* < 0.005 versus A*β* group.

**Figure 4:**
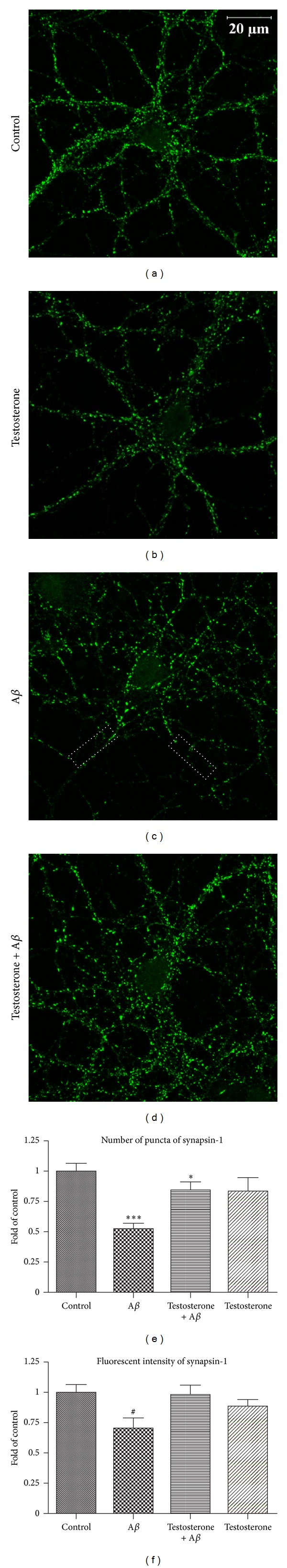
Testosterone attenuated oligomeric A*β*-induced reduction of synapsin-1 in primary hippocampal neurons. Primary hippocampal neurons were treated with 10 nM testosterone for 1 h, followed by exposure to 5 *μ*M oligomeric A*β* for 24 h. Neurons were stained with synapsin-1 antibody. (a) Control, (b) 10 nM testosterone for 25 h, (c) 5 *μ*M A*β* for 24 h, and (d) 10 nM testosterone for 1 h, followed by exposure to 5 *μ*M A*β* for 24 h. (e) The number of puncta and (f) the fluorescent intensity were analyzed by Image J software as described in the above section. ***P < 0.0005 versus control group, ^#^ *P* < 0.05 versus control group, and *P < 0.05 versus A*β* group. White dot boxes indicate reduced number of puncta along neurites.

To study the effects of testosterone against A*β*-induced damage on neurites, immunostaining of microtubule-associated protein-2 (MAP-2) was performed. Exposure of hippocampal neurons (DIV 14) to A*β* caused fragmentation of neurites and reduced their length (Figure 1(c)) when compared to the control (Figure 1(a)). Pretreatment of neurons with testosterone for 1 h attenuated the damaging effects of A*β* on neurites. As shown in Figure 1(d), there were less fragmented neurites and the architecture of neurites has been preserved. In order to examine the protective effects of testosterone against A*β* in synaptic regions, immunocytochemical staining of synaptic vesicle proteins including synaptophysin, synaptotagmin, and synapsin-1 was conducted. As shown in Figures 2(a), 3(a), and 4(a), synaptophysin, synaptotagmin, and synapsin-1 were shown as fine puncta along neurites in the control groups, respectively. The number of puncta and fluorescent intensity of synaptophysin, synaptotagmin, and synapsin-1 were markedly decreased after A*β* treatment (Figures 2(c), 3(c), and 4(c)). The toxic effects of A*β* were reversed by exposing hippocampal neurons to testosterone for 1 h (Figures 2(d), 3(d), and 3(d)). Statistical analysis showed that the pretreatment of testosterone significantly elevated A*β*-induced reduction of the number of puncta and fluorescent intensity of synaptophysin and synaptotagmin (Figures 2(e), 2(f), 3(e), and 3(f)). Testosterone also provided protection on synapsin-1, although it is not statistically significant. All these data suggested that testosterone reduced A*β*-mediated synaptic damage and it preserved synaptic vesicle proteins.

### 3.2. Testosterone Reversed Oligomeric A*β*-Induced Exocytosis Dysfunction

**Figure 5:**
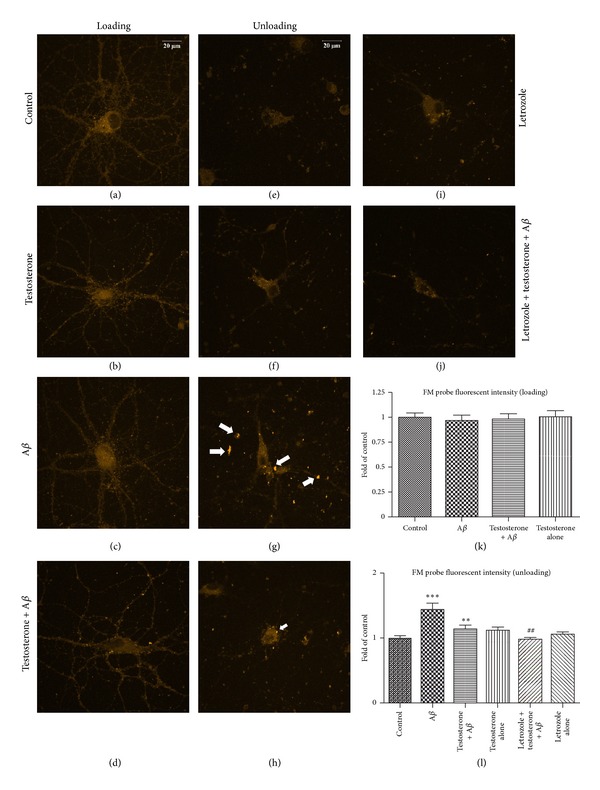
Oligomeric A*β*-induced impairment of synaptic vesicle unloading was ameliorated by pretreatment of testosterone. Primary hippocampal neurons were treated with testosterone or letrozole (aromatase inhibitor) + testosterone, followed by exposure to 5 *μ*M oligomeric A*β* for 24 h. Neurons were stained with FM4-64 fluorescent probe. (a)–(d) represent synaptic vesicle uptake FM probe capability. (a) Control, (b) testosterone 10 nM for 25 h, (c) A*β* 5 *μ*M for 24 h, and (d) 10 nM testosterone for 1 h, followed by exposure to 5 *μ*M A*β* for 24 h. (e–j) represent synaptic vesicle release FM probe capability. (e) Control, (f) testosterone 10 nM for 25 h, (g) A*β* 5 *μ*M for 24 h, (h) 10 nM testosterone for 1 h, followed by exposure to 5 *μ*M A*β* for 24 h, (i) Let 1 *μ*M for 25 h, (j) treatment of 1 *μ*M Let, and 10 nM testosterone for 1 h, followed by exposure to 5 *μ*M A*β* for 24 h. (k) The statistical analysis of FM probe loading fluorescent intensity and (l) the fluorescent intensity of FM probe unloading were measured by Image J software as described in the above section. ***P < 0.001 versus control group, **P < 0.01 versus A*β* group, and ^##^ *P* < 0.01 versus A*β* group.

Neurotransmission within synapses can be affected by membrane receptors, ion channels, endocytosis, and exocytosis. In this study, FM fluorescent probe was applied to the neurons to examine the loading and unloading function of synaptic vesicles. The fluorescent probe was successfully uptaken by synaptic vesicles in all groups (Figures 5(a)–5(d)). There was no significant difference in fluorescent intensity between A*β*-treated group and control group in FM probe loading experiment (Figure 5(k)), suggesting that endocytosis was not affected by A*β* peptide.

On the other hand, the neuroprotective effects of testosterone were revealed in FM probe unloading experiment. Aggregation of the FM probe was found in A*β*-treated neurons (Figure 5(g)), suggesting that A*β* caused impairment of the exocytosis process, and hence FM probe could not be released. However, when neurons were exposed to testosterone for 1 h prior to A*β*, the aggregation of FM probe was significantly reduced compared with those without testosterone (Figure 5(h)). To investigate the neuroprotective effects of testosterone after blocking of the estrogenic pathway, an aromatase antagonist letrozole (1 *μ*M) was used for further experiments [37]. When neurons were exposed to testosterone and letrozole for 1 h followed by exposure to A*β* for 24 h, FM probe fluorescent intensity was also significantly lower than that of the A*β*-treated group (Figure 5(l)). The results suggested that A*β*-induced exocytosis dysfunction could be restored by testosterone probably through an estrogenic-independent pathway.

### 3.3. Testosterone Retained Heat Shock Protein in Neurons under A*β* Insults

**Figure 6:**
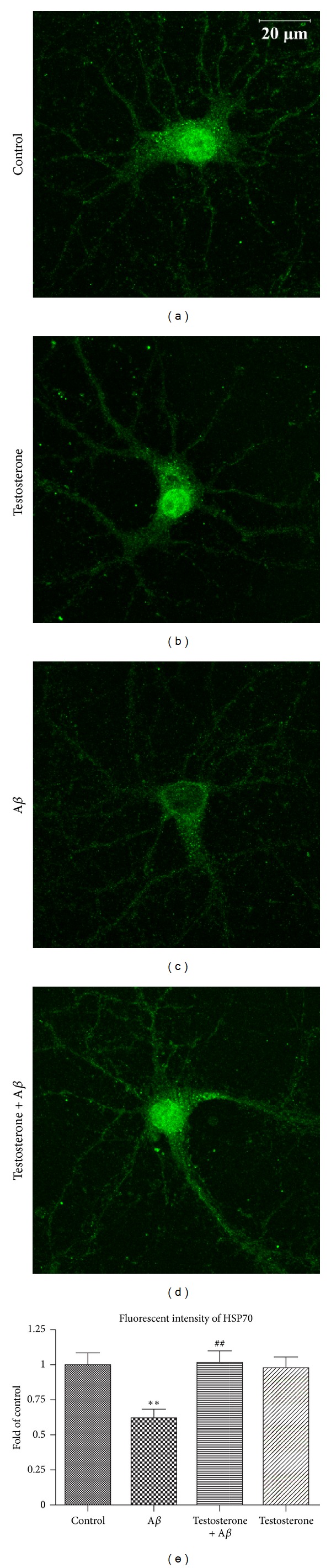
Testosterone attenuated oligomeric A*β*-induced reduction of heat shock protein in primary hippocampal neurons. Primary hippocampal neurons were treated with 10 nM testosterone for 1 h, followed by exposure to 5 *μ*M oligomeric A*β* for 24 h. Neurons were stained with HSP70 antibody. (a) Control, (b) 10 nM testosterone for 25 h, (c) 5 *μ*M A*β* for 24 h, and (d) 10 nM testosterone for 1 h, followed by exposure to 5 *μ*M A*β* for 24 h. (e) The fluorescent intensity was analyzed by Image J software as described in the above section. **P < 0.01 versus control group; ^##^ *P* < 0.01 versus A*β* group.

Heat shock proteins are molecular chaperones that play important regulatory roles for the stabilizing and even facilitate the clearance misfolded or aggregated proteins via chaperone-mediated autophagy. This is particularly important for the turnover of presynaptic proteins. The fluorescent intensity of HSP70 was tremendously decreased in A*β*-treated neurons when compared with neurons in the control group (Figures 6(a) and 6(c)). For neurons exposed to testosterone prior to A*β*, the fluorescent intensity of HSP70 was markedly restored (Figure 6(d)). Analysis of images showed that the level of HSP70 in testosterone treated group was even similar to the control group (Figure 6(e)).

## 4. Discussion

The male sex hormone testosterone has demonstrated its neuroprotective effects in various studies [38–40]. In AD, testosterone has been shown to improve neuronal viability and reduce A*β* accumulation and AD-like pathological changes in animal and cell culture models [34, 41]. Based on these findings, we are interested to further explore the potential protective effects of testosterone against A*β* neurotoxicity. Specifically, we aim to examine its effects on oligomeric A*β*-induced synaptic changes. We found that testosterone preserved cytoskeletal protein (MAP-2) and synaptic vesicle proteins in A*β*-treated hippocampal neurons. Testosterone also mitigated A*β*-induced synaptic exocytosis dysfunction probably through an estrogenic independent pathway. In addition, we showed that testosterone treatment attenuated A*β*-induced reduction of HSP70. Our data suggest that testosterone is effective in reducing A*β*-induced synaptic dysfunction in rat hippocampal neuron, and this may explain the importance of physiological dosages of testosterone in the prevention of AD.

Testosterone is known to modulate synaptic plasticity and is involved in the maturation of spine [42, 43]. It has been proved that the hormone can reach the brain via blood circulation, or it can be synthesized locally in the hippocampus in a low yet sufficient concentration to modulate synaptic density and synaptic functions [44, 45]. In our study, we have shown that physiological level of testosterone was able to attenuate devastating changes in the synapse. It is interesting to note that testosterone mainly attenuated changes in the presynaptic compartment (preserving synaptophysin, synaptotagmin, and synapsin-1) from our results. Since we did not detect obvious changes between the control and A*β*-treated groups for postsynaptic proteins such as postsynaptic density 95 (PSD-95) (Supplementary Figure 1, see Supplementary Material available online at http://dx.doi.org/10.1155/2014/103906), we did not further investigate the effects of testosterone on the postsynaptic proteins. In the study conducted by Ziehn and colleagues, it was found that testosterone could restore the excitatory synaptic transmission and the levels of both pre- and postsynaptic proteins in a mouse model of multiple sclerosis (experimental autoimmune encephalomyelitis, EAE) [46]. They reported that testosterone restored the levels of PSD-95 during EAE and attenuated the atrophy of hippocampus. In another study conducted by Li and colleagues, testosterone replacement in castrated mice also restores the decreased level of PSD-95 [42]. These studies suggested that testosterone may preserve the postsynaptic compartment (spine) and it is likely to have an effect on PSD-95. It is not clear why testosterone could not preserve oligomeric A*β*-induced changes on PSD-95 in our model. Since the two studies were conducted in animals in which microglia were present to surround neurons, it is therefore possible that testosterone has an anti-inflammatory role to mediate its action on PSD-95 through monitoring the neighboring microglia [46]. In fact, in our* in vitro* model, testosterone is likely to have direct effects on neurons because the growth of microglia was minimized in our preparation protocol.

Testosterone can bind to androgen receptors, or it can be aromatized locally to estradiol and then bind to estrogen receptors [17]. We found that the application of letrozole to block the conversion of testosterone to estradiol did not repress the protective effects of testosterone against oligomeric A*β*, as indicated in our FM probe exocytosis experiment. The results suggested that it is unlikely for testosterone to elicit its neuroprotective benefits through activation of estrogen receptors. In fact, our data showed that cotreatment of testosterone and letrozole further attenuated A*β*-induced reduction in FM probe fluorescent intensity. The results suggest that testosterone* per se* is able to revert impairment of presynaptic functions. The next question is how testosterone modulates different presynaptic proteins. Classical signaling for testosterone to exert its effects is to bind to androgen receptor. This may display different heat shock proteins (HSP), especially HSP70 and HSP90.

HSP70 and HSP90 are molecular chaperone which regulates the folding of proteins and recognizes misfolded polypeptides. HSP are closely related to the pathogenesis of neurodegenerative diseases including AD and Parkinson's disease. It was shown in a pilot study that the protein level of HSP70 was markedly decreased in the olfactory receptor neurons of subjects with AD when compared to age-matched controls [41]. In another postmortem brain study, the expression of HSP70 was significantly increased in the temporal cortex of patients with AD [47]. These findings suggest that the level of HSP70 might change with stress.* In vivo* study supported the protective role of HSP70 in synapse. The induction of HSP70 prior to heat shock preserved synaptic performance deficit from subsequent hyperthermia insult [48]. Moreover, overexpression of HSP70 could suppress AD-related phenotypes and reduce synaptic loss in a transgenic AD mice model [49], further indicating the importance of HSP70 during AD pathogenesis. In our study, we had demonstrated that A*β*-induced decrease in HSP70 protein level was restored in the testosterone treatment group. This finding is in line with those reported by Zhang and colleagues, showing that testosterone and estrogen attenuated neuronal cell death induced by intracellular A*β*_1–42_. They reported that testosterone treated group has increased levels of HSP70 and comicroinjection of HSP70 with A*β*_1–42_ blocks the neurotoxicity of the peptide [27]. The molecular mechanism underlying HSP70 neuroprotection against oligomeric A*β* toxicity is not clear. Previous studies suggested that HSP70 could prevent the translocation of p53 to the nucleus, thus inhibiting apoptosis [47, 49]; yet it is still uncertain if this is involved in the observed synaptic changes. One possible explanation for the observed improvement in synaptic vesicle release would be the stabilizing effects of HSP70 on presynaptic protein expression. In fresh pond water snails, early induction of HSP70 stabilized presynaptic proteins (syntaxin I, synaptic vesicle protein 2, and synaptotagmin I) expression and attenuated hypoxia-induced motor and sensory impairment [50]. HSP70 may also confer protection to the presynaptic compartment by stabilizing calcium concentration and calcium influx. It has been reported that HSP70 can interact with the cytosolic loop of CaV2.3 R-type voltage-gated calcium channel [51]. The interaction of HSP70 and calcium channel might be important for the prevention of calcium overload during stress condition, thus contributing to better synaptic transmission [52].

In summary, we have shown that physiological concentrations of testosterone protect hippocampal neurons from oligomeric A*β*-mediated synaptic toxicity and in the same time increase the expression of HSP70 in these neurons. Given that the use of testosterone replacement therapy is still controversial with safety concern [53], understanding the downstream effectors of testosterone in the AD brain may be helpful for the identification of potential pathway specific neuroprotective agents.

## Supplementary Material

Supplementary Figure 1: Oligomeric A*β*-induced did not reduce the expression of PSD-95 in primary hippocampal neurons. Primary hippocampal neurons were exposed to 5 *μ*M oligomeric A*β* for 24 h. Neurons were stained with PSD-95 antibody. (A) Control, (B) 5 *μ*M A*β* for 24 h. Representative photos were captured by Carl Zeiss LSM-510Meta/Axiocam inverted confocal microscope.
