# Supplementary material for: Protective Effects of Testosterone on Presynaptic Terminals against Oligomeric β-Amyloid Peptide in Primary Culture of Hippocampal Neurons
Source: Biomed Res Int. 2014 Jun 18;2014:103906. doi: 10.1155/2014/103906 (PMC4086619; doi:10.1155/2014/103906)
Supplement: Supplementary file 1 — Supplementary Figure 1: Oligomeric Aβ-induced did not reduce the expression of PSD-95 in primary hippocampal neurons. Primary hippocampal neurons were exposed to 5 μM oligomeric Aβ for 24 h. Neurons were stained with PSD-95 antibody. (A) Control, (B) 5 μM Aβ for 24 h. Representative photos were captured by Carl Zeiss LSM-510Meta/Axiocam inverted confocal microscope. [file 103906.f1.pdf]

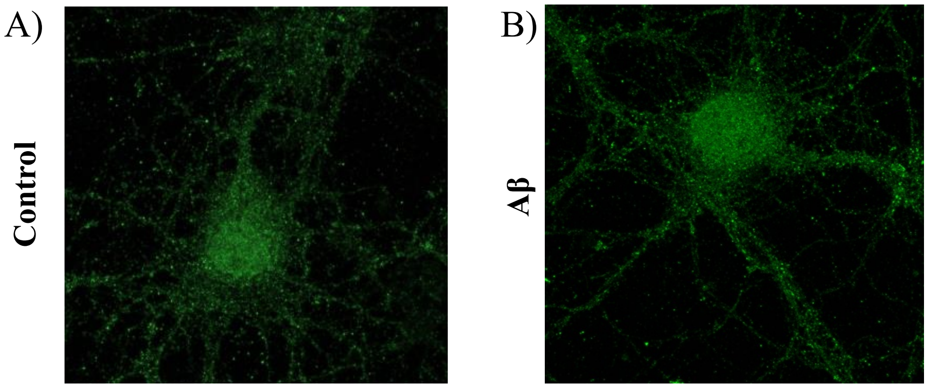

Supplementary Figure 1 **Lau et al., 2014**

Supplementary Figure 1. Oligomeric Aβ did not induce changes in the expression of PSD95 in primary hippocampal neurons. Primary cultures of hippocampal neurons were exposure to 5 μM oligomeric Aβ for 24 h. Neurons were stained with PSD95 antibody. (A) Control, (B) 5 μM Aβ for 24 h.
